# Impaired Axonal Regeneration in Diabetes. Perspective on the Underlying Mechanism from *In Vivo* and *In Vitro* Experimental Studies

**DOI:** 10.3389/fendo.2017.00012

**Published:** 2017-02-01

**Authors:** Kazunori Sango, Hiroki Mizukami, Hidenori Horie, Soroku Yagihashi

**Affiliations:** ^1^Diabetic Neuropathy Project, Department of Sensory and Motor Systems, Tokyo Metropolitan Institute of Medical Science, Tokyo, Japan; ^2^Department of Pathology and Molecular Medicine, Hirosaki University Graduate School of Medicine, Hirosaki, Japan; ^3^TechnoMaster Co. Ltd., Yokohama, Japan

**Keywords:** diabetic neuropathy, axonal regeneration, animal models, dorsal root ganglia, Schwann cells

## Abstract

Axonal regeneration after peripheral nerve injury is impaired in diabetes, but its precise mechanisms have not been elucidated. In this paper, we summarize the progress of research on altered axonal regeneration in animal models of diabetes and cultured nerve tissues exposed to hyperglycemia. Impaired nerve regeneration in animal diabetes can be attributed to dysfunction of neurons and Schwann cells, unfavorable stromal environment supportive of regenerating axons, and alterations of target tissues receptive to reinnervation. In particular, there are a number of factors such as enhanced activity of the negative regulators of axonal regeneration (e.g., phosphatase and tensin homolog deleted on chromosome 10 and Rho/Rho kinase), delayed Wallerian degeneration, alterations of the extracellular matrix components, enhanced binding of advanced glycation endproducts (AGEs) with the receptor for AGE, and delayed muscle reinnervation that can be obstacles to functional recovery after an axonal injury. It is also noteworthy that we and others have observed excessive neurite outgrowth from peripheral sensory ganglion explants from streptozotocin (STZ)-diabetic mice in culture and enhanced regeneration of small nerve fibers after sciatic nerve injury in STZ-induced diabetic rats. The excess of abortive neurite outgrowth may lead to misconnections of axons and target organs, which may interfere with appropriate target reinnervation and functional repair. Amelioration of perturbed nerve regeneration may be crucial for the future management of diabetic neuropathy.

## Introduction

**Figure 1:**
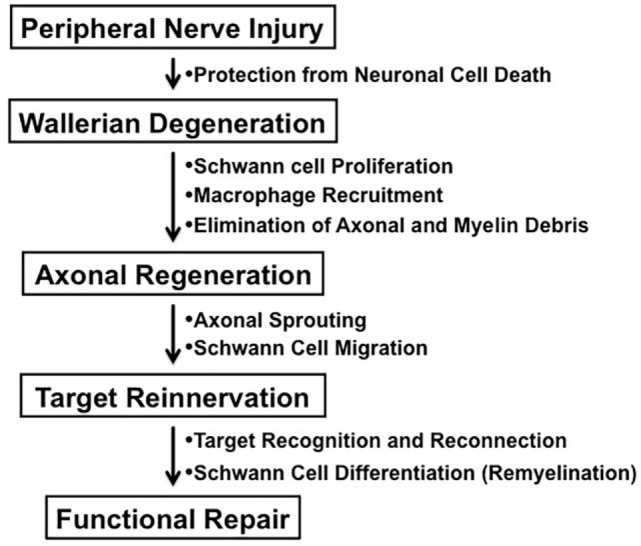
**The process of peripheral nerve regeneration after injury**.

In contrast to the unsuccessful axonal regeneration in the central nervous system (CNS) in adult mammals, neurons in the peripheral nervous system (PNS) have a potential to grow new axons after injury. Successful nerve regeneration with functional recovery in the PNS can be attributed to both intrinsic and extrinsic factors, such as *the ability of neurons and/or Schwann cells to regenerate neurites, the distal environment supportive of axon regrowth*, and *the target tissues receptive to reinnervation* (Figure 1) (1). Although growth inhibitory molecules, such as chondroitin sulfate proteoglycans and myelin-associated glycoprotein, are present in both adult CNS and PNS, Wallerian degeneration of the peripheral nerves distal to the injury provides an optimal environment for axonal sprouting from the injured neurons (1, 2). The sequence of cellular events during Wallerian degeneration includes Schwann cell activation and proliferation, macrophage recruitment, elimination of axonal and myelin debris, and synthesis of neurotrophic and chemotactic factors. However, axonal regeneration and reinnervation depends on appropriate contact of regenerating axonal sprouts with Schwann cell basal laminae in the distal nerve segment. Currently, clinical approaches to repair axonal injury are still far from satisfactory (3).

Diabetic peripheral neuropathy is one of the most common and intractable complications of diabetes mellitus, and its prevalence correlates closely with the degree and duration of the disease (4). Impaired axonal regeneration is one of the salient features of diabetic neuropathy; however, its pathogenesis remains to be elucidated (5). In this paper, we review the findings from recent studies that focus on how diabetes affects the intrinsic and extrinsic factors of peripheral nerve regeneration. In addition, we discuss our own results from *in vivo* and *in vitro* studies, which indicate that defective neurite outgrowth after axonal injury occurs under diabetic conditions (6–8).

## Impaired Axonal Regeneration in Animal Models of Diabetes

**Figure 2:**
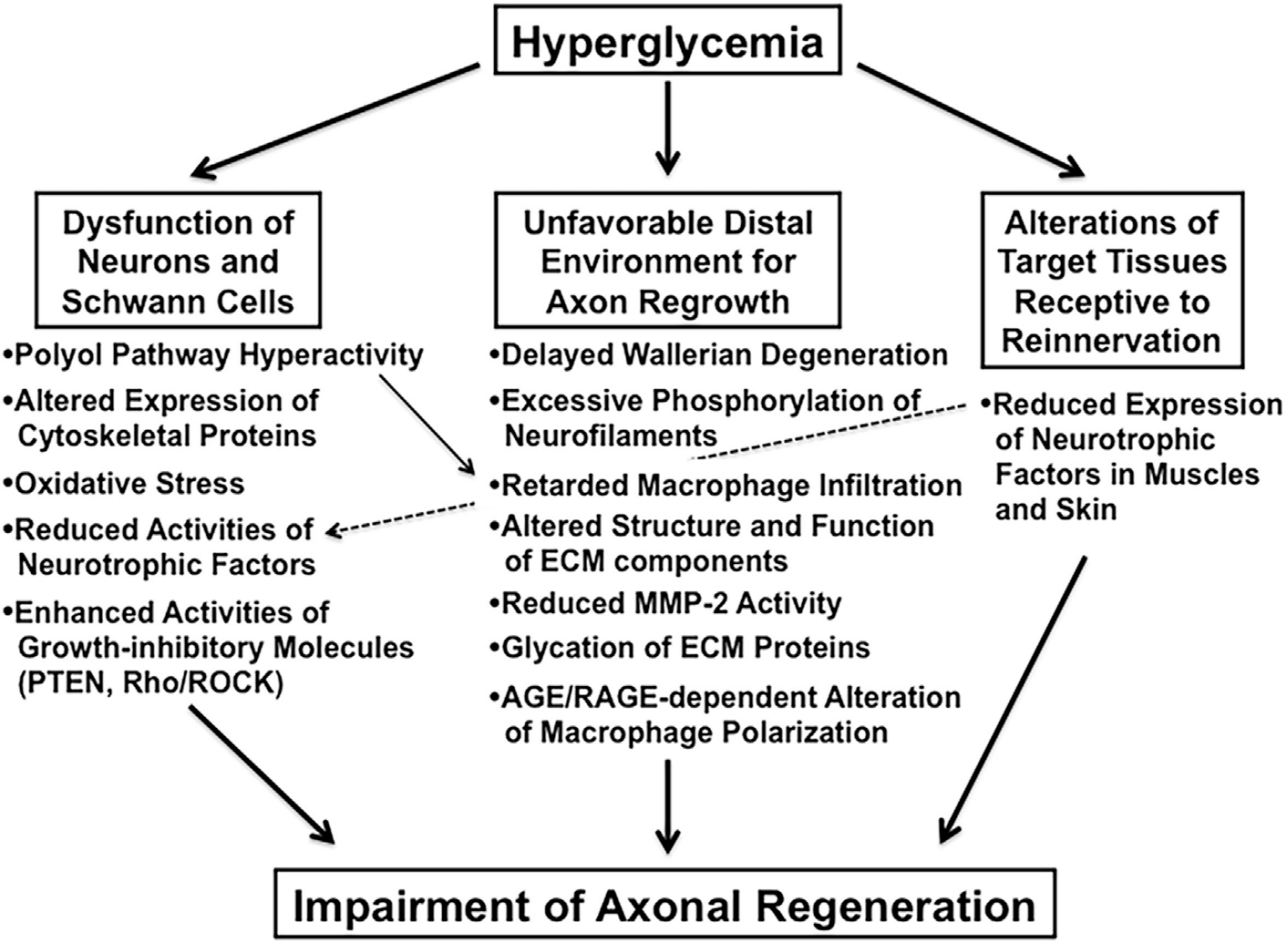
**The suggested abnormalities leading to impaired axonal regeneration in diabetic animals**.

Impaired axonal regeneration following peripheral nerve injury is a characteristic feature of diabetic neuropathy and has been documented in experimental diabetic animals [e.g., streptozotocin (STZ)-diabetic rats (9) and mice (10), biobreeding Worcester (BB/Wor) rats (11), and Goto-Kakizaki rats (12)]. We classify these animal studies into the three categories indicated in italics in Section “Introduction” and briefly introduce recent studies focusing on the abnormalities in each category (Figure 2).

### Altered Biological Properties of Neurons and Schwann Cells for Axonal Regeneration in Diabetes

Alterations in the biological properties of neurons and Schwann cells under diabetic conditions are likely to affect neurite outgrowth (5, 13, 14). So far, polyol pathway hyperactivity (11), altered tubulin and neurofilament expression (15), oxidative stress (16), and reduced synthesis/transport of neurotrophic factors (17, 18) have been implicated in the deficient axonal regeneration in diabetic animals. In addition to those studies, upregulation of growth inhibitory molecules in diabetic neurons and/or Schwann cells has recently attracted much attention. Phosphatase and tensin homolog deleted on chromosome 10 (PTEN) is an intrinsic inhibitor of phosphoinositide-3-kinase Akt signaling pathway and has been suggested to negatively regulate axonal regeneration in the CNS (19) and PNS (20). PTEN is predominantly expressed in small IB4-binding dorsal root ganglion (DRG) neurons, axons, and Schwann cells (20). Its neuronal expression is significantly elevated in STZ-diabetic mice relative to that in normal littermates (21). Moreover, knockdown of PTEN in DRG by non-viral retrograde short interfering RNA uptake was shown to accelerate electrophysiological recovery of the peripheral nerve and myelinated axonal regrowth as well as skin reinnervation after sciatic nerve crush injury in diabetic mice (21). These findings imply that suppression of PTEN activity can be a novel strategy for restoration of the regenerative capacity of diabetic neurons.

The small G protein Rho and its downstream target Rho kinase (ROCK) regulate cell adhesion, migration, and proliferation through actin cytoskeleton assembly and cell contraction (22). Rho and ROCK are recognized as potent inhibitors for axonal regeneration (23) and inactivation of Rho/ROCK has been shown to enhance axonal regeneration (24, 25). A recent study by Kanazawa et al. (26) suggested an involvement of Rho/ROCK in the pathogenesis of diabetic neuropathy. In their study, RhoA and ROCK activity in the sciatic nerve was enhanced in STZ-diabetic rats, and treatment with fasudil, a specific ROCK inhibitor, ameliorated reduced nerve conduction velocities in diabetic rats. In addition, several studies with rodent models of diabetes have argued for the efficacy of Rho/ROCK inhibitors against painful signs (27) and erectile dysfunction (28). However, there has been no direct evidence that Rho/ROCK inhibition restored deficient axonal regeneration in diabetic animals. Exendin-4 (Ex-4), a glucagon-like peptide 1 (GLP-1) receptor agonist, is now widely used for patients with type 2 diabetes because of its glucose-dependent insulinotropic actions and somatostatin-dependent glucagon inhibition. In addition to such pancreatic actions, direct actions of GLP-1 mimetics on the nervous system have been a matter of interest. Ex-4 promoted neurite outgrowth of PC12 cells and adult rodent DRG neurons (29–31) and restored the neurological abnormalities of STZ-diabetic mice without normalizing blood glucose levels (30). Suppression of RhoA activity (31) and an inverse relationship between RhoA activity/expression and neurite outgrowth activity or viability of DRG neurons (32, 33) may be implicated in the mechanisms accounting for the neuroprotective effects of Ex-4.

### Unfavorable Distal Environment for Axon Regrowth

Following peripheral nerve injury, the distal ends of axons and myelin sheaths undergo Wallerian degeneration that is a prerequisite for the subsequent axonal regrowth and reinnervation to the target tissues (5, 34). A delay in the process of Wallerian degeneration appears to be closely associated with impaired axonal regeneration in diabetic animals (11, 35). Excessive phosphorylation of neurofilaments in peripheral nerves can be a cause of delayed Wallerian degeneration in STZ-diabetic rats (35) because phosphorylated neurofilaments are resistant to degradation by proteases activated by axonal injury. Removal of axonal and myelin debris by activated Schwann cells and recruited macrophages contributes to the guidance of the pathway for axonal regeneration. Therefore, impaired activities of these cells in response to nerve injury under diabetic conditions may also be implicated in delayed Wallerian degeneration (10, 36). According to Chen et al. (37), retarded macrophage infiltration and impaired vascularization in the distal stump of the transected sciatic nerves was ameliorated in diabetic mice with AR deficiency (AR^−/−^). As a consequence, diabetic AR^−/−^ mice exhibited improved Wallerian degeneration and nerve regeneration with functional recovery relative to that in diabetic AR^+/+^ mice. These findings are consistent with the beneficial effects of AR inhibitors (ARIs) that restored nerve regeneration after injury in diabetic animals (11, 38). They also suggest the involvement of polyol pathway hyperactivity in the deficit of macrophage-associated process in Wallerian degeneration. However, given the fact that AR is predominantly expressed in Schwann cells and epineurial endothelial cells rather than macrophages in the PNS (39), it remains unclear how the increased polyol flux leads to impaired recruitment of macrophages.

The extracellular matrix (ECM) of the peripheral nerve provides scaffold for the cellular components to modulate their properties through interactions between the cell-surface receptors (e.g., integrins) and their specific ligands on the ECM. Major ECM proteins, such as collagens, laminin, and fibronectin, have been shown to promote axonal regeneration *in vivo* (40) and *in vitro* (13). Chondroitin sulfate proteoglycans are recognized as repulsive substrata for neurons in the PNS (41, 42). Thus, diabetes-induced alterations in the structure and function of the ECM components in the PNS appear to intervene the regenerative responses of nerve fibers. Matrix metalloproteases (MMPs) are a family of zinc-dependent proteolytic enzymes responsible for the degradation and remodeling of ECM components (5). In the PNS, MMP-9 expression is rapidly upregulated at the sites of axonal injury and appears to have a major role in Wallerian degeneration. On the other hand, MMP-2, which expression is upregulated at later stages within the outgrowing neurites, may provide a more permissive environment for regeneration by removing inhibitory ECM molecules, such as chondroitin sulfate proteoglycans (43, 44). Ali et al. (45) recently reported downregulation of the activity and expression of MMP-2 in the sciatic nerve of STZ-diabetic rats relative to that of normal rats. In addition, reduced neurite outgrowth of cultured DRG neurons on sciatic nerve cryosections from diabetic rats was partially rescued by pretreatment with active MMP-2. These findings suggest that impaired axonal regeneration in diabetes is attributable in part to the unfavorable microenvironment caused by reduced MMP-2.

A significant increase in the content of advanced glycation endproducts (AGEs) in endoneurial ECM proteins was detected in chronic STZ-diabetic rats (46). Glycation of ECM proteins under exposure to long-term hyperglycemic conditions may affect cell–matrix interactions and make the proteins more resistant to protease digestion, thereby leading to impaired axonal regeneration (46, 47). Receptor for AGEs (RAGE) is a multiligand receptor of the immunoglobulin superfamily and interacts with not only AGEs but also various kinds of proinflammatory and/or regulatory molecules, such as the S100 family of cytokines, high-mobility group box 1 protein, amyloid beta-peptide, lysophosphatidic acid, and mammalian diaphanous-1 (48). In the PNS, RAGE is predominantly expressed in Schwann cells and endothelial cells and upregulated in diabetes (49, 50). It is also recognized that RAGE expression becomes prominent in activated Schwann cells and infiltrating macrophages upon acute peripheral nerve injury (51). Juranek et al. (52) recently reported that RAGE-deficient diabetic mice exhibited improved axonal regeneration after sciatic nerve crush compared to diabetic wild mice. In their study, diabetes-induced decreases in the number of endoneurial blood vessels and regenerating neurites at the crush side were significantly restored in diabetic mice deficient of RAGE. They further investigated macrophage infiltration into the nervous tissue distal to the site of injury in RAGE-deficient diabetic mice. The population of proinflammatory type M1 macrophages was significantly higher in diabetic wild-type mice than in non-diabetic ones; however, RAGE-deficient diabetic mice exhibited a decrease in the population of M1 macrophages and an increase in anti-inflammatory type M2 macrophages relative to those in diabetic wild-type mice. On the basis of these findings, the researchers hypothesized that impaired axonal regeneration in diabetic mice is, at least in part, attributed to RAGE-dependent alteration of macrophage polarization. In agreement with this finding, treatment of bone marrow-derived macrophages with AGEs promoted M2 macrophage polarization into the M1 phenotype through the activation of RAGE/nuclear factor-κB pathway (53). In contrast, however, pharmacological inhibition of RAGE by soluble RAGE or F(ab′)_2_ fragments of antibodies raised to RAGE or its ligands suppressed axonal regeneration after sciatic nerve crush in non-diabetic mice (54). Other studies have also suggested that RAGE *per se* is a positive modulator for axonal regeneration in the PNS (55, 56). It remains to be solved why RAGE inactivation has such contrasting effects on axonal regeneration between the hyperglycemic and normoglycemic conditions. The RAGE ligands other than AGEs, in particular, S100B, and high-mobility group box 1 have been suggested to activate the RAGE signaling pathway in Schwann cells and macrophages to promote their migration to injured sites, thereby contributing to axonal regeneration with functional repair (51, 57–59). Under diabetic conditions, however, the AGE–RAGE signaling axis that is detrimental to axonal regeneration may surpass the non-AGE-mediated RAGE signaling axes described above.

### Alterations of Target Tissues Receptive to Reinnervation

Reinnervation of the peripheral target tissues is the final stage of axonal regeneration. Atrophy and denervation in the target tissues disturb functional repair even if regenerating axons successfully reach their targets (1). A body of evidence indicates that the impaired axonal regeneration in diabetes can be attributed largely to the derangement of neurons and Schwann cells and the unfavorable environments for axonal sprouting and elongation as described above. There are several studies, however, on diabetes-induced alterations of target tissues receptive to reinnervation. Homs et al. (60) reported that muscle reinnervation was delayed in both non-obese diabetic (NOD) mice and STZ-diabetic mice following sciatic nerve crush, but the changes were more robust in NOD mice than in STZ mice. Reduced expression of neurotrophin-3 in muscles is suggested to be a cause of insufficient reinnervation and muscle weakness in diabetes (61). In addition, prosaposin-derived peptide TX14(A) restored nerve regeneration deficits and muscle denervation atrophy after sciatic nerve crush in STZ-diabetic rats. In this setting, TX14(A) may exert direct effects on muscles in response to nerve injury rather than nerve regenerative capability (62). Additionally, reduced production of neurotrophic molecules in diabetic skin may also affect epidermal reinnervation after axonal injury (63, 64).

## Excessive Neurite Outgrowth from Ganglion Explants in STZ-Diabetic Mice

**Figure 3:**
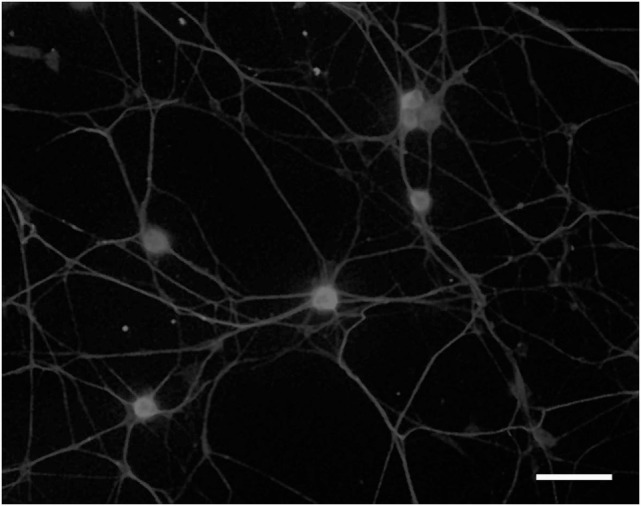
**Dissociated cell culture of adult rat dorsal root ganglion neurons**. Cells are fixed and immunostained with anti-βIII tubulin antibody. Bar = 100 µm.

**Figure 4:**
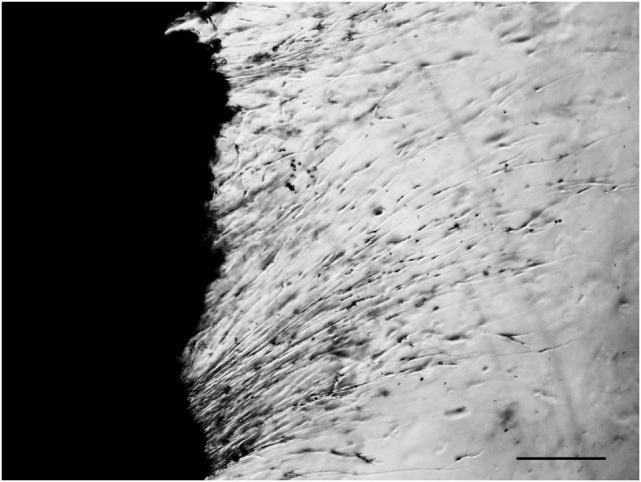
**Explant culture of adult mouse dorsal root ganglion**. Neurites are elongating from transected nerve terminals. Bar = 100 µm.

In 1991, we introduced cell culture system of dissociated DRG neurons from experimental diabetic animals (Figure 3) (65, 66). Since then, this system has been recognized as a useful tool to explore the changes of neurons that are exposed to hyperglycemia for a significant period of time and is now widely employed for the study of diabetic neuropathy (13, 67). However, it remains controversial whether the neurite outgrowth of diabetic DRG neurons is impaired, and the cell culture system of dissociated DRG neurons has the following problems for evaluation of axonal regeneration. First, there is a possibility that severely damaged neurons in diabetic animals are eliminated during the procedure of culture. Second, there is a topographic difference in the site of neurite outgrowth where neurites sprout from neuronal cell bodies *in vitro*, whereas axonal regeneration occurs at the sites of injury *in vivo*. Third, neurons are separated from other neurons and non-neuronal cells. It is thus likely that the effects of cellular interplay on axonal regeneration cannot be evaluated by this system. To overcome these issues, we have established a three-dimensional culture system of ganglion explants in which adult peripheral ganglia with nerve fibers are embedded in collagen gel or Matrigel^®^, and neurite outgrowth from transected nerve stump has been evaluated (Figure 4) (13, 68). Because of the maintenance of cell-to-cell interactions, it may be reasonable to consider that the explant culture mimics axonal regeneration *in vivo* better than the use of dissociated cells. We introduced this culture system to vagal nodose ganglia and DRG of STZ-diabetic mice and found that the neurite outgrowth from transected nerve stump in diabetic mice was not diminished but rather *enhanced* (6, 7). These findings were contrary to our expectations and in stark contrast to those in previous studies *in vivo*. Since it is well accepted that hyperglycemia impairs neural function, enhanced neurite outgrowth *in vitro* in our study does not seem to imply the acceleration of proper axonal regeneration after injury in diabetes. Considering that the explant culture lacks the process of Wallerian degeneration and subsequent regenerative events at the site distal to injury, it seems more likely that the excess of regenerating fibers in diabetes may impede appropriate target reinnervation and functional recovery. This hypothesis was supported by the findings of *in vivo* studies (5, 8, 69), which will be discussed in the next section. Gumy et al. (36) applied the explant culture system to normal adult mice and observed that exposure to high-glucose (60 mM) culture conditions resulted in retardation of neurite outgrowth and Schwann cell migration from DRG explants. These findings are in disagreement with those in our studies (6, 7) and may be accounted for by the differential effects of long-term (>3 weeks) hyperglycemia *in vivo* in our study versus short-term (<1 week) high-glucose environments *in vitro* on neurite outgrowth in their study. In neither study, however, the hyperglycemic insults induced apoptotic cell death of DRG neurons. These findings indicate that apoptosis (70, 71) is unlikely to be a major contributor to the impaired axonal regeneration in diabetic neuropathy.

## Increased Small-Fiber Density after Nerve Injury in STZ-Diabetic Rats

Using electron microscopy, Yasuda et al. (38, 69) evaluated the regenerative capacity of myelinated nerve fibers (MNFs) and unmyelinated nerve fibers (UNFs) in STZ-diabetic and normal control rats at 5 and 24 weeks after sciatic nerve crush. The number and size of regenerating MNFs after crush injury were significantly decreased in diabetic rats relative to those in control rats. The structural changes were consistent with the reduced motor nerve conduction velocity detected in diabetic rats with nerve crush (38). In contrast, the density and number of UNFs were markedly increased in both normal and diabetic groups at 5 weeks after injury, and these parameters returned to their baseline levels in normal control rats but not in diabetic rats at 24 weeks after injury (69). Although the precise mechanisms accounting for the differential effects of hyperglycemia on the regenerative capacity of MNFs versus that of UNFs remain unclear, the authors inferred that the long-lasting impairment of regenerative capacity of UNFs in diabetes might disturb the proper connection between the nerve endings and target organs. This inference seems to be well fitted with our hypothesis based on the *in vitro* assays (6, 7). Moreover, they found amelioration of the reduced motor nerve conduction velocity and number and size of regenerating MNFs in the crushed nerves in diabetic rats treated with tolrestat, an ARI (38). There were no significant effects, however, on the regenerative capacity of UNFs in the ARI-treated diabetic rats (69). ARI may therefore be more effective for regeneration of MNFs with functional repair than UNFs under diabetic conditions, and the effects may be dependent on the high expression of AR in the Schwann cells (39, 72).

By light microscopic and ultrastructural morphometric analyses, we recently evaluated nerve fiber regeneration from axotomized sciatic nerves of STZ-diabetic rats and non-diabetic control rats (8). Four weeks after axotomy, there was a significant increase in myelinated fiber density and total fiber number at the cut end in diabetic rats relative to those in control rats. In contrast, the average number of myelin lamellae per fiber, measured by electron microscopy equipped with a computer-assisted image analyzing system, was significantly reduced in diabetic rats relative to that in control rats. These findings indicate enhanced regeneration of thinly MNFs after axotomy in rats with experimental diabetes. Impaired myelination of regenerated fibers may be attributable to metabolic disorders of Schwann cells and increased vulnerability of the myelin sheath to oxidative stress under diabetic conditions (73–75). Increased regeneration of small nerve fibers in this study may be associated with the improper target reinnervation described above and painful phenotypes of diabetic neuropathy (76), but further evidence is required to support this contention.

## Translational Findings in Patients with Diabetic Neuropathy

Although the purpose of this paper is to overview altered axonal regeneration in experimental diabetes, several findings illustrated here are closely associated with those from clinical studies. For instance, the restorative effects of ARIs on MNF regeneration in diabetic rats (11, 38) were also indicated in the specimens of sural nerve biopsies from patients with diabetic neuropathy (77, 78). Exogenously applied neurotrophic factors, growth factors, and other biological molecules improved axonal regeneration and other neurological functions in animal models of diabetes (5, 17, 18, 79); however, the translation of these molecules (e.g., NGF, BDNF, and VEGF) to humans has failed in clinical trials (80–82). These failures may be attributable to the irrelevance of animal models and outcome measures to the pathology and pathophysiology of human diabetic neuropathy (83).

Because the loss of corneal nerves has been shown to correlate well with the severity of peripheral nerve damage, corneal confocal microscopy (CCM) is recognized as a novel non-invasive diagnostic modality for patients with diabetic neuropathy (84). CCM has also been applied to animal models of diabetes, and Yorek et al. (85) reported that glycemic control by continuous administration of insulin in STZ-diabetic mice significantly restored corneal nerve innervation. In agreement with this study, long-term normoglycemia by simultaneous pancreas and kidney transplantation in patients with type 1 diabetes improved corneal nerve fiber density, branch density, and length (86). In contrast, the transplantation failed to ameliorate the loss of epidermal nerve fiber density that was evaluated by skin biopsies (87). These findings imply that CCM can be a more sensitive means than skin biopsies for the assessment of early nerve regeneration and repair after therapeutic interventions.

## Conclusion

Diabetes mellitus has been shown to affect peripheral nerve regeneration in various ways. In the first part of this paper, we reviewed the topics of axonal regeneration in animal models of diabetes from the viewpoint of intrinsic and extrinsic factors, in particular, focusing on the events that occurred at the sites distal to the injury. Delayed Wallerian degeneration, alterations in the structure and function of the ECM components, and enhanced AGE–RAGE signaling axis caused by hyperglycemia can be major obstacles to axonal regrowth after injury. In the second part, we illustrated the excessive regenerative capacity of small nerve fibers under diabetic conditions *in vivo* and *in vitro* (6–8, 69). There is room for further investigation if these findings imply *dysfunctional* neurite outgrowth that disturbs appropriate target reinnervation and functional recovery. In addition, much more attention should be paid to the diabetes-induced abnormalities of target tissues receptive to reinnervation.

## Author Contributions

All the authors designed and performed the experiments described in this paper in accordance with the ethical guidelines in their institutions. KS and SY wrote the paper.

## Conflict of Interest Statement

The research was conducted in the absence of any commercial or financial relationship that could be construed as a potential conflict of interest.
